# Bloodstream Infection and Its Clinical Characteristics and Relevant Factors Associated with Interventional Therapy in a Large Tertiary Hospital: A Six Years Surveillance Study

**DOI:** 10.1155/2019/8190475

**Published:** 2019-10-21

**Authors:** Yanling Bai, Zhigang Zheng, Mingmei Du, Hongwu Yao, Yunxi Liu, Jijiang Suo

**Affiliations:** ^1^Department of Infection Management and Disease Control, Chinese PLA General Hospital, Fuxing Road No. 28, Beijing 100853, China; ^2^The First Outpatient Department of General Logistics Department, Fuxing Road No. 22, Beijing 100842, China

## Abstract

**Background:**

Interventional therapy has been widely used in the medical field as its advantages of minimally invasive, safe and quick recovery. Bloodstream infection (BSI) is the most common healthcare-associated infections (HAIs) after interventional therapy, but there are few reports about it. This study intends to analyze the clinical characteristics and relevant factors of BSI after six years of interventional therapy in a large tertiary teaching hospital, in order to provide guidances for the prevention and control of BSI after interventional operations.

**Methods:**

The case information of patients with BSI after interventional therapy from 2013 to 2018 were collected through the "real-time monitoring system of healthcare-associated infections". All BSI was determined by the infection control full-time staff and clinicians. Questionnaires were designed to review case by case and register the relevant patient information into a database. A total of 18 relevant factors were counted. Statistical software was used for analysis.

**Results:**

174 cases of BSI occurred in 25401 patients, the incidence was 0.69%, and BSI accounted for 50% of all infected sites. Gram-positive bacteria accounted for 56.05%, coagulase-negative Staphylococcus was the main infectious bacteria. Relevant risk factor analysis showed that hepatocellular carcinoma, had undergone surgery, biliary complications, prophylactic antibiotic, replacement of antibiotics, number of interventional operations, days of prophylactic antibiotic use were the related risk factors associated with BSI (*P* < 0.05). Multivariate analysis showed that days of prophylactic antibiotic use (OR = 1.586, *P* < 0.05) and replacement of antibiotics (OR = 13.349, *P* < 0.05) were the main risk factors associated with the development of BSI.

**Conclusions:**

BSI is the main infection site after interventional surgery. For patients with the risk factors as hepatocellular carcinoma/biliary complications/had undergone surgery etc., the time of prophylactic antibiotic use can be prolonged properly before interventional surgery, and selection of single antibiotic appropriate for use could significantly aid preventive measures to avoid occurrence of BSI.

## 1. Introduction

Interventional therapy, with its advantages of minimally invasive, safe and rapid recovery, has been rapidly developed since its application in medical treatment in the 1970s, especially in the diagnosis and treatment of hepatocellular carcinoma (HCC) and hepatobiliary diseases [1]. Under the condition of no exposure to the lesion, it uses catheters, guide wires and other devices to carry out minimal trauma treatment for some lesions under the guidance of imaging devices (X-ray, ultrasound, CT, MRI) [2]. However, many invasive operations such as puncture, intubation, and drug injection need to be performed during interventional operation, and extended length of stay in hospital environment after operation provides a way for pathogenic bacteria to invade. Therefore, Healthcare-associated Infections (HAIs) is the most common complication [3–5]. According to the research, the incidence of Bloodstream infection (BSI) in patients with HCC after angiography is 4.0% [6], and after embolization is 2.7% [7]. Therefore, it is important to find out the risky factors of BSI in time and give effective treatment as soon as possible for the rehabilitation of patients after operation.

To our knowledge, this type of study focused on the analysis of the infection situation of specific treatment methods for a certain disease. There were few and old studies on specific infection sites (such as BSI) [4–8]. This study analyzed the clinical characteristics and relevant risk factors of BSI after interventional therapy in a large hospital from 2013 to 2018, aiming at providing guidances for prevention and control of BSI.

## 2. Materials and Methods

### 2.1. Materials

From January 1, 2013 to December 31, 2018, a total of 25401 inpatients in a tertiary hospital in Beijing in China were collected. 174 of them suffered from BSI. According to inclusion exclusion criteria, 119 patients were included in this study which as the infection group, including 99 males and 20 females, with an average age of 59.08 ± 12.30 years. According to the same sex, same department, same age (±2), same operation time (±2w), 1 : 1 matched uninfected patients, there were another 119 cases which served as control group. This study was approved by the Medical Ethics Committee.

#### 2.1.1. Inclusion Criteria

(1) Interventional surgery performed in this hospitalization; (2) According to the diagnostic or clinical criteria, BSI was confirmed by clinical symptoms, signs and pathogenic bacteria examination; (3) Complete case data.

#### 2.1.2. Exclusion Criteria

(1) No operation in this hospitalization; (2) BSI before operation; (3) Patients had multiple hospitalizations and repeated BSI during the study; (4) Combine community infection; (5) Blood culture was identified as contamination by diagnostic criteria.

#### 2.1.3. Diagnostic Criteria for BSI

According to CDC diagnostic criteria for BSI in the United States [9]: Laboratory BSI diagnosis should meet the criteria as follows: (1) Blood culture is positive once or more, and the positive pathogen has nothing to do with other infected sites or can be sure coming from other infected sites because of the same bacteria detected. (2) The patient has at least one of the following symptoms: fever (>38℃), shivering or hypotension. At least one of the following items can be satisfied: (1) If the blood culture is a common skin microorganism, the blood culture should be positive twice or more at different times. (2) If the blood culture is a common skin bacteria and the blood culture is positive only once, the same pathogen which is positive by venous catheter culture is needed. (3) Blood antigen test was positive (e.g., *Haemophilus influenzae*, *Streptococcus pneumoniae*, *Neisseria meningitidis*, or Group B streptococcus), and the symptoms, signs and laboratory results could not be explained by infection in other parts.

#### 2.1.4. Blood Culture Contamination Criteria

It should meet at least one of the following criteria [10, 11]: (1) No obvious fever and risk factors (such as low immune function or invasive operation); (2) Despite the above risk factors, many subsequent blood cultures proved to be other pathogens; (3) Ineffective empiric antibiotic therapy; (4) Fever can be explained by other reasons such as immunity of tumors, and no obvious signs of infections. (5) Laboratory standards, such as extended incubation of blood culture positive; continuous multiday culture only once for bacteria; blood culture once to isolate more than two kinds of skin flora.

### 2.2. Methods

Through the “real-time monitoring system of healthcare-associated infections” to collect the case information of patients with BSI in interventional department during the research period, design questionnaires, using them after evaluation by experts, checking the cases one by one in detail, and register the patients' gender, age, basic diseases, disease diagnosis, time of admission and exit, type of operation, past history, and occurrence of BSI (date, pathogenic bacteria, etc.), time and type of antibiotic prophylaxis use, etc. Total 18 relevant factors were counted.

### 2.3. Statistical Analysis

Differences in categorical variables were assessed using Pearson *χ*^2^ test or Fisher's exact test (when expected cell frequencies were <5). Paired nominal-scale data were used *t*-Test. Multivariate Analysis were used multi-variate logistic analysis. SPSS version 19.0 was used for all statistical analyses. A two-tailed *P* value of <0.05 was considered to be statistically significant.

## 3. Results

### 3.1. Incidence of BSI

Total 25401 patients were monitored in 6 years, and 348 cases (including lower respiratory infection 51 cases, surgical site infection 34 cases, urinary tract infection 25 cases, BSI 174 cases, upper respiratory infection 19 cases, alimentary infection 17 cases, etc.) were infected. Among them, 174 cases (accounted for 50.0%) had BSI, and the overall incidence of BSI was 0.69%. From 2013 to 2018, with the increase of the number of hospitalized patients, the incidence of BSI also increased significantly, but with no statistic difference (*χ*^2^ = 9.770, *P* > 0.05) (Table 1).

### 3.2. Pathogenic Bacteria

157 pathogenic bacteria were detected from 119 patients. The majority (88, 56.05%) were Gram-positive bacteria. The most prevalent isolates were Coagulase-negative *Staphylococcus* (CNS) (48, 30.57%) and *Escherichia coli* (32, 20.38%). Total 116 drug-resistant bacteria were detected from 157 (73.88%) (Table 2). 

### 3.3. Description of Clinical Characteristics

Analysis of the clinical data of the infected cases, showed that the majority of the patients were males, accounting for 83.19%. Most of them were over 60 years old (55.46%). 38.66% of them had combined basic diseases. 41.18% of the patients had complications with acute or chronic biliary diseases before operation. 43.70% of the patients had undergone surgical resection of primary tumors. The average incidence of BSI was 2.5 ± 2.306 times, the first time intervention was the most common, accounting for 47.90%. The average incidence of BSI was 3.02 ± 2.393 days, accounting for 53.78%. Primary BSI accounted for 74.79%, secondary BSI accounted for 10.08%, and transient bacteremia accounted for 15.13%. HCC was assessed as the main risk factor associated with infection (87.39%). Transarterial chemoembolization (TACE) was the main risk associated procedure (46.22%).

Among them 98.32% of the patients had used antibiotics before the operation. The total prophylactic antibiotic usedays were 3.01 ± 2.444. The highest used antibiotic grade was restricted antibiotics (55.46%), Cephalosporin was the most commonly used antibiotics classification (70.08%), the second was Quinolones (5.13%). 23.08% of the patients used antibiotics jointly, the major combined was Cephalosporin and Nitroimidazoles. 13.68% of the patients had changes in type of antibiotic treatment during the prophylactic use (Table 3).

### 3.4. Relevant Risk Factors Analysis

Sex, age, weight, multiple clinical diseases, primary diseases, surgical procedures, surgical staff, frequency of chemotherapy, frequency of interventional operations, biliary complications, had undergone surgery, time of prophylactic antibiotic use, types. etc. 18 indicators were included in *χ*^2^ test or *t*-test. The results showed that 6 indicators were *P* < 0.05, respectively: disease diagnosis, had undergone surgery, biliary complications, prophylactic antibiotic use, replacement of antibiotics, number of interventional operations and days of prophylactic antibiotic use (Table 4).

### 3.5. Multivariate Analysis

Six meaningful indicators of single factor analysis were included in logistic analysis. Variables were screened step by step by the forward method. The regression equation was fitted with the retention condition *P* < 0.05. The factors that finally entered the fitting model were: days of prophylactic antibiotic use, and replacement of antibiotics (Table 5).

## 4. Discussion

With the rapid development of interventional surgery in the medical field, it is used more and more widely in clinic. With the increasing numbers of operations, it is particularly important to effectively control HAIs and improve medical quality [1, 2]. China has a high incidence area of hepatocellular carcinoma. At present, the incidence and mortality rate of hepatocellular carcinoma accounts for 50.0% worldwide. Surgical resection and recurrence are the most widely used and effective options. As a minimally invasive technique, interventional chemotherapy for HCC directly acts on the lesion by introducing corresponding chemotherapeutic drugs and biological agents through catheters. The goal is more accurate, and local effective concentration is higher, and also it is safer and more effective than systemic chemotherapy [12, 13]. Although it is recognized as a safe treatment, various complications such as self-limiting postembolization syndrome, biloma, BSI, hepatic failure, cholecystitis, gastrointestinal bleeding, pancreatitis, renal failure, liver infarction, and liver abscesses have been reported [14–16]. According to the data of our study, the overall incidence of BSI was 0.69%, accounting for 50% of the total infection sites, most of the BSI patients were HCC patients receiving TACE, accounting for 46.22%. BSI had become the major type of infection after interventional surgery in a hospital. The occurrence of BSI has aggravated the medical burden and seriously affected the prognosis of the disease. This needs further attention and study.

A total of 157 strains of bacteria were detected in blood culture, of which 88 were Gram-positive bacteria (56.05%), 68 were Gram-negative bacteria (43.31%) and 1 was fungus (0.64%). It was reported that Gram-negative bacilli had the highest detection rate of BSI [17]. However, in recent years, with the wide application of broad-spectrum antibiotics and the increase of indwelling catheters and invasive operations, the pathogenic structure of BSI has changed greatly, and the detection rate of Gram-positive cocci has gradually increased [18]. Relevant studies in the United States and Europe had reported that more than 65.0% of BSI was caused by Gram-positive bacteria. Coagulase-negative *Staphylococcus* (CNS), *Staphylococcus aureus* and *Enterococcus* were the most common pathogens in BSI [19]. CNS is a weak virulent bacterium represented by *Staphylococcus epidermidis* and *Staphylococcus hemolyticus*. Before 1970s, CNS was often regarded as a contaminant bacterium in clinic [20]. However, in recent years, monitoring CNS has accounted for 1–3^rd^ of BSI in hospital [21]. 72 cases CNS were detected in this study. On the basis of strict application of the diagnostic criteria, 24 cases of CNS with suspected contamination were eliminated. The contamination rate of CNS was 33.3%. The hospital strictly required multiple samples of blood to reduce the contamination rate as much as possible, but there were still instances of blood culture contaminations. Infection control preventive measures including appropriate choice of disinfection products, cleaning and disinfection surgical procedure protocols and blood culture collection protocols must be implemented consistently in accordance to current regulatory guidelines. A total of 48 CNS (54.55%) causing BSI were obtained after eliminating contamination. Among them, 39 oxacillin-resistant coagulase-negative staphylococci were detected, the detection rate was 81.25%, slightly higher than the rate above 70% be reported by Ledha A [22]. It is suggested that CNS is the most important bacteria in BSI, and the drug resistance is emerging as serious complication of antibiotic therapy. Although the United States reported [23] that *Staphylococcus aureus* accounted for 20.0% of BSI in hospitals were the most important Gram-positive pathogenic bacteria, but only two strains of *Staphylococcus aureus* were isolated in our study, and none of them were resistant to antibiotics. It was worth noting that 9 strains of Micrococcus (7 strains of drug-resistant) and 9 strains of drug-resistant Gram-positive bacilli were also detected in this group, and the infection caused by them was transient asymptomatic bacteremia.

*Escherichia coli* had been detected 32 strains (47.06%) which was the dominant strain of gram-negative bacilli. It was reported that the clinical detection rate of *Escherichia coli* in BSI is 18.7% [23]. The production of Extended-Spectrum Beta-Lactamases (*ESBLs*) was the main resistance mechanism of *Escherichia coli*, and its detection rate continues its increasing incremental rise. The positive rate of *ESBLs* was 27.8%–30.1% [19,  23]. In our study 14 of the 32 strains of ESBLs accounted for 43.75%. 7 positive were for ESBLs in another 10 strains of *Klebsiella pneumoniae*, and five strains (3 strains were resistant) were detected in 2018, which was consistent with the increase of detection rate of Carbapenem-resistant *Klebsiella pneumoniae* in recent two years [24]. Increasing concern about virulence of KPC requires greater attention and further study.

Male patients were mainly infected in this group of data, which was 3 times compared with females, with no statistical significance; the patients who were older than 60-years-old and with HCC were the majority (55.46%). The infection rates of HCC and other diseases were significantly different (*P* < 0.05), which was related to the infection of the biliary tract system caused by liver adjacent to the biliary tract system, puncture and drainage, or the patients complicated with biliary tract slowness. Bacteremia risk factors associated with had biliary complications (41.18% in this study, *P* < 0.05), puncture of blood vessels to break the infected bile into the blood system [25]. 46.22% of the infected patients received perfusion and chemoembolization, and the necrotic liver tissue after embolization or chemoembolization was other major risks associated with infection, while the blood supply artery of liver tumors was minimized during the operation. At the same time, it will lead to insufficient blood supply to the intrahepatic bile duct, which will eventually lead to bile duct injury and the infection of biliary pathogens in the liver tissue [26]. Gram-negative bacteria are thought to be mostly associated with infections through this channel. In addition, whether or not had undergone surgery was performed the related factor of BSI (*χ*^2^ = 10.717, *P* < 0.05). This study also concluded that the number of interventional operations was related to the incidence of infection (*P* < 0.05). The average number of interventional operations occurred in patients with 2.5 ± 2.306 interventions, with the first interventional operation accounting for 47.90%. Whether this is related to the body's initial stress response needs further study. Among 119 cases of BSI, 89 cases were primary BSI, accounting for 74.79%, which occurred within 48 h after interventional operations, accompanied by fever, chills and inflammatory factors elevated. 12 cases were secondary BSI, accounting for 10.08%, 11 cases were second to surgical site infection, 1 case was second to lower respiratory infection. 18 cases were transient bacteremia, accounting for 15.13%, which had only a transient fever and recovered without the replacement of antibiotic for therapy.

Because of the high rates of HAIs in interventional surgery, antibiotics should be used routinely, but some studies have shown that prophylactic antibiotic before interventional surgery can not prevent the occurrence of infection [27]. Pathogenic bacteria can still be cultured from the blood, there are several possibilities: inadequate dosage of antimicrobial agents; irregular medication time; potential for lapse or break in aseptic technique during surgical procedures [28]. In these data, 98.32% of the patients had used antibiotics before operation, 88.89% were given antibiotics for the first time in 0.5–2 hours before operation. The highest used antibiotic grade was restricted antibiotics (55.46%), Cephalosporins was the most commonly used antibiotics classification (70.08%), Cefmetazole and Cefuroxime were the main drugs, but there was no statistical difference in both use level and class of antibiotics (*P* > 0.05). 23.08% of the patients used antibiotics jointly and 13.68% of them changed antibiotics during preventive use. The total prophylactic antibiotic days were 3.01 ± 2.444, 67.22% of the patients were treated with a higher level of antibiotics immediately after infection. Compared with the control group, the overall incidence of BSI in the replacement of antibiotics was higher (*P* < 0.05), and the overall time of prophylactic antibiotic use in the infected group was shorter than that in the control group (*P* < 0.05). These two points were the independent risks of infection. We suspect that inadequate prophylactic antibiotic or replacement of antibiotics may be the main cause of BSI after interventional surgery. Therefore, this study suggests the associated risk factors of hepatocellular carcinoma biliary complications, had undergone surgery, etc., the timing of antibiotic prophylaxis including proper length of antibiotic administration and appropriate antibiotic selection to single antibiotic class to avoid unnecessary antibiotic therapy with multiple changes and replacement in therapy prior to and after surgical procedure.

However suggested changes in prophylactic antibiotic therapy cannot replace standard infection control measures of cleaning and disinfection and sterilization of equipment and instrumentation. Preventive actions to strengthen the perioperative environment, defined cleaning and disinfection protocols, post-operative protocols and other infection control and antibiotic stewardship based measures can reduce the incidence of HAIs after surgical procedures.

## Figures and Tables

**Table 1:** Occurrence of BSI in interventional patients in different years.

Year	No. of patients	No. of HAIs	No. of BSI	Proportion %	Rate %
2013	3911	51	24	47.06	0.61
2014	4013	51	19	37.25	0.47
2015	4155	48	22	45.83	0.53
2016	4160	54	28	51.85	0.67
2017	4577	63	40	63.49	0.87
2018	4585	81	41	50.62	0.89
Total	25401	348	174	50.00	0.69

**Table 2:** Detection of pathogenic bacteria in blood culture.

Pathogenic bacteria	No. (%) of bacteria	No. (%) of drug-resistant bacteria
Gram-positive bacteria	88/157 (56.05%)	64/88 (72.73%)
Coagulase-negative *Staphylococcus*	48/157 (30.57%)	39/48 (81.25%)
*Micrococcus luteus*	9/157 (5.73%)	7/9 (77.78%)
*Enterococcus faecium*	14/157 (8.92%)	7/14 (50.0%)
Gram-positive bacilli	9/157 (5.73%)	9/9 (100.0%)
*Enterococcus faecalis*	4/157 (2.55%)	1/4 (25.0%)
*Staphylococcus aureus*	2/157 (1.27%)	0
Others	2/157 (1.27%)	1/2 (50.0%)
Gram-negative bacteria	68/157 (43.31%)	32/68 (47.06%)
*Escherichia coli*	32/157 (20.38%)	14/32 (43.75%)
*Klebsiella pneumoniae*	10/157 (6.37%)	6/10 (60.0%)
*Enterobacter cloacae*	8/157 (5.10%)	3/8 (37.5%)
*Pseudomonas aeruginosa*	6/157 (3.82%)	1/6 (16.67%)
*Acinetobacter baumannii*	3/157 (1.91%)	3/3 (100.0%)
Others	9/157 (5.73%)	5/9 (55.56%)
Fungus	1/157 (0.64%)	0
*Candida glabrata*	1/157 (0.64%)	0

Note: definition of drug-resistant of major bacteria, (1) Oxacillin-resistant coagulase-negative Staphylococci, (2) methicillin-resistant *Staphylococcus aureus*, (3) carbapenem resistant *Escherichia coli*, (4) carbapenem resistant *Klebsiella pneumonia.*

**Table 3:** Clinical characteristics of patients with BSI after interventional surgery.

Variables	Grouping, No (%) of cases	
Gender	Male, 99 (83.19%)	Female, 20 (16.81%)
Age	≤40,10 (8.4%)	41~60, 43 (36.13%), ≥60, 66 (55.46%)
Combined basic diseases	Yes, 46 (38.66%)	No, 73 (61.34%)
Biliary complications	Yes, 49 (41.18%)	No, 70 (58.82%)
Had undergone surgery	Yes, 52 (43.70%)	No, 67 (56.30%)
No. of interventional operations	once, 57 (47.90%)	No. 2~5, 52 (43.70%), No. ＞5, 10 (8.40%)
The first positive blood culture day after operation	≤2 days, 64 (53.78%)	3–5 days, 42 (35.29%), >5 days, 13 (10.92%)
BSI property	Primary BSI, 89 (74.79%)	Secondary BSI, 12 (10.08%), Transient bacteremia, 18 (15.13%)
Disease diagnosis	HCC, 104(87.39%)	Others, 15(12.61%)
Term of operation	Microwave ablation, 38 (31.93)	TACE, 55 (46.22%), RFCA, 14 (11.76%), Others, 12(10.08%)
Prophylactic antibiotic use	Yes, 116 (98.32%)	No, 3 (1.68%)
Use of antibiotics on the day of operation	Yes, 103 (86.55%)	No, 16 (13.45%)
Antibiotic grade	Unrestricted, 4 (36.79%)	Restricted, 66 (55.46%), Special, 7 (5.88%)
Days of prophylactic antibiotic use	1 day 44 (36.79)	2~5 days, 61 (51.26%), ＞5 days, 14 (11.76%)
Combined prophylactic antibiotic use	Yes, 27 (23.08%)	No, 90 (76.92%)
Replacement of antibiotics	Yes, 16 (13.68%)	No, 102 (87.18%)

Note: RFCA = Radiofrequency Ablation; (1) Primary BSI as blood culture is positive once or more, and the positive pathogen has nothing to do with other infected sites; (2) Secondary BSI as blood culture is positive once or more, and the positive pathogen can confirm come from other infected sites. Because they have detected the same bacteria; (3) Transient bacteremia as blood culture is positive once or more but without any symptoms.

**Table 4:** Relevant factors of BSI in patients undergoing interventional surgery.

Variables	Experimental group (*N* = 119)	Control group (*N* = 119)	*χ* ^2^	*P*
Disease diagnosis				
HCC	104	81	15.801	0.001
Others	15	38		
Had undergone surgery				
Yes	53	29	10.717	0.001
No	66	90		
Biliary complications				
Yes	49	23	13.461	0.001
No	70	96		
Prophylactic antibiotic use				
Yes	117	96	19.710	0.001
No	2	23		
Replacement of antibiotics				
Yes	16	4	5.697	0.019
No	100	92		
No. of interventional operations	2.5 ± 3.880	1.96 ± 1.729	5.833	0.04
Days of prophylactic antibiotic use	3.01 ± 2.444	4.73 ± 2.469	0.031	0.01

**Table 5:** Multivariate logistic of BSI in patients undergoing interventional surgery.

Variables	*B*	S.E	Wals	*P*	OR	95% CI
Days of prophylactic antibiotic use	.461	.090	26.240	.001	1.586	1.329~1.892
Replacement of antibiotics	2.591	.925	7.846	.005	13.349	2.177~81.835

## Data Availability

The data used to support the findings of this study are available from the corresponding author upon request.
